# Sports Metaresearch: An Emerging Discipline of Sport Science and Medicine

**DOI:** 10.1007/s40279-025-02181-x

**Published:** 2025-04-01

**Authors:** John Warmenhoven, Paolo Menaspà, David N. Borg, Simine Vazire, Nicole White, Kristin Sainani, Sophia Nimphius, Aaron J. Coutts, Franco M. Impellizzeri

**Affiliations:** 1 School of Sport, Exercise and Rehabilitation, Faculty of Health & University of Technology Sydney (UTS), Human Performance Research Centre, University of Technology Sydney (UTS), Moore Park, NSW Australia; 2 Australian Sports Commission, Australian Institute of Sport, Bruce, ACT Australia; 3 School of Exercise Nutrition Sciences, Queensland University of Technology, Brisbane, QLD Australia; 4 Melbourne School of Psychological Sciences, University of Melbourne, Parkville, VIC Australia; 5 Australian Centre for Health Services Innovation, School of Public Health and Social Work, Queensland University of Technology, Brisbane, Australia; 6 Division of Epidemiology, Department of Health Research and Policy, Stanford University, Stanford, USA; 7 School of Medical and Health Science, Edith Cowan University, Joondalup, WA Australia

## Abstract

Inadequacies in the conduct and quality of research are well established across many research domains, including sport science and medicine. Metaresearch—the practice of performing research on research—is presented as a practical vehicle for improving research quality through evaluating the research processes. This article introduces the concept of metaresearch to sport as a new sub-field of sport science. The broad types of metaresearch are introduced, with a mapping of current sports metaresearch activity across these areas. Interdisciplinary centres aimed at improving scientific quality across other fields are also introduced to sport, and specific considerations for beginning metaresearch are provided for sport. This includes, for example, not performing metaresearch poorly, beginning evaluative metaresearch early to intervene before bad practice becomes normalised, leveraging required interdisciplinary expertise depending on the metaresearch question and undertaking an ethical approach for carrying out evaluation of research quality.

## Key Points

**Table:** 

Metaresearch plays an important function in the evaluation and monitoring of research, which highlights potential problems with the literature base within a field and provides the research community with the ability to make better informed judgements on the implementation of study findings.
Metaresearch can also be used as a vehicle for identifying specific issues that are present within disciplines of research, which can then be used to develop specific solutions through integration with other fields of research and disciplines.

## Introduction

Inadequacies in the conduct and quality of research are well established across most research domains [1–3], including inadequately powered studies, lack of validation and replication of findings and problems associated with researcher and publication bias. In medicine, issues with the statistical analysis of studies and their conclusions were identified as early as 1966 [4], with concerns being identified with the conclusions drawn from medical research due to the application of appropriate statistical methods. The most prominent article was published in 1994 by Altman, with the provocative albeit true title *The Scandal of Poor Medical Research* [5]. Altman noted that many errors found across experimental medical research were likely a consequence of a pressure to publish, and subsequent research being conducted by individuals without adequate training. In the late 2000s, it was estimated that approximately 85% of global spending on health and medical research resulted in research waste [6] and that more than 15 years after the report by Altman, poor research remained a serious concern [7].

### Research Waste and Misapplication of Statistics in Sport

There has been a similar emergence of broader issues regarding the quality and utility of sports science and sports medicine research. These include methodological issues such as application of flawed statistical methods that are not subjected to expert statistical review. A well-known example is the widespread use of magnitude-based inference [8, 9]. This statistical method was proposed and introduced in sports research with good intentions. However, this was a statistical method developed within sport with error rates that vary widely, with type 1 error rates often being unacceptably high for making robust conclusions in experimental sport research [8, 10]. More recently, other issues have become apparent. Sport researchers have created their own analytical frameworks for individual responder analyses [11], and biomechanists have developed their own analytical frameworks for performing hypothesis testing on curves representing biomechanical variables [12]. In each of these instances, conventional statistical methods were either modified or misapplied, leading to research studies built on questionable theoretical underpinnings and findings. Being able to identify these types of errors in the poor application of statistics in sport research is important to understand the extent to which studies overstate positive research outcomes. This behaviour contributed to a biased evidence base as a source of research waste.

### Poor Reporting in Sport Science and Medicine

In addition to the misapplication of statistical methods, research on scientific practice in sport has also identified issues with experimental design and poor reporting. Issues with statistical power in between-subject designs [13], sample size calculations for reliability studies [14, 15], and reporting of information for reproducing power analyses [16] have been noted in motor behaviour research. There are also issues related to bias in the reporting of research outcomes in sport science and sports medicine. Mesquida et al. [17] discussed the potential presence and potential consequences of publication bias, questionable research practices (QRP), including p-hacking, and issues with studies with underpowered designs on the replicability of published findings in sport. Büttner et al. [18] and Twomey et al. [19] alluded to the possibility of issues with reporting bias, demonstrating that the proportion of supported hypotheses in sports medicine has been reported as implausibly high. Borg et al. [20] then provided preliminary evidence of this bias, in the reporting of ratios. Borg et al. [21] also demonstrated issues related to the handling and reporting of missing data across a sample of papers in sport science and medicine. Identifying issues related to scientific practice is also essential for understanding whether there are likely to be issues related to publication bias in subdisciplines of sport research, prior to any interventions or new findings being used in practice.

### Fraud in Sport Science

Beyond issues with the quality of reporting, there are examples of potentially fraudulent research in sport. Improbable data patterns have been identified in multiple papers within strength and conditioning [22]. Additionally, there are often issues related to the omission of important information as a part of the publication process. Norris et al. [23] identified that open data, materials, analysis and replication attempts are currently rare in physical activity behaviour change intervention studies, with these issues also being mirrored by Schulz et al. [24], who showed issues in the availability of information on blinding, randomisation and other experimental design factors.

### Difficulties with Applied Research in Sport

General issues in conducting science in sport also have the potential to become more likely, due to the *unusualness* of how sport research can be carried out, particularly in high performance sport settings. Often, in high performance environments, science is undertaken by practitioners/scientists embedded in the daily training environment to assist in decision making, so that research and sports science and sports medicine support can become intertwined. In these contexts, many practitioners (i.e., scientists and clinicians) working with Olympic, Paralympic and professional sporting programs start their practitioner careers on a research pathway [25]. This context can lead to a subsequent blurring of the lines between collecting and using data descriptively for monitoring and decision making quickly with athletes, versus carefully planning and conducting science for the purposes of conducting quality research in sport. Additionally, there can be pressure to maintain academic relevance, and continue publishing while working as an early career scientist in sport, given the large degree of cross-over between academia and high-performance sport for people with specialist expertise [25].

The entanglement of research and practice has likely evolved organically, as a function of opportunity, given the importance of preserving the high-performance sport environment while answering research questions. The embedding of research into high-performance practitioner environments serves to produce more generalisable outcomes that can be used by coaches and athletes within their daily training environments. Coaches are likely to be more accepting of sport science or medicine research if the research can be demonstrated to have a clear impact (and by inference direct relevance) within their own practice environment [26]. Despite some advantages, this has the potential to lead to substantial problems in the application of research methods in sporting environments that are not well designed nor intended for carefully controlled research studies. As an example, in these circumstances high rates of participant drop-out are common, due to challenges that make it difficult to fit testing for research into the regular training and competition program, or due to issues with athletes receiving injuries, subsequently leading to smaller sample sizes in sport. Additionally, participant drop-out may affect the whole testing group at different testing points in a study design if there are changes to a whole team for competition scheduling or other sport demands, leading to study designs being compromised and data collection plans deviating from the original research plan.

### A Need for Formalisation of Metaresearch

In terms research volume, sports science and sports medicine already have literature in some disciplines spanning multiple decades [27–30], presenting a large body of evidence that may already inform practice and policy. There is a need to formalise a type of research that assists in the evaluation of these existing bodies of evidence, so that outcomes from sport can be evaluated before being applied safely and ethically in practice environments. This type of research is often referred to as metaresearch. Additionally, given that sport often incorporates characteristics of the high-performance environment into different research questions and research designs, it is possible that metaresearch could be used to highlight areas where issues exist in scientific practice. Metaresearch can therefore be viewed as an opportunity to partner with the right expertise to progress sport science and medicine forwards.

## What is Metaresearch?

Metaresearch is research on research itself. The concept of performing research to better understand scientific processes has existed long before metaresearch, in the form of fields such as history of science, philosophy of science and sociology of science [31]. In contrast, metaresearch applies a more practical lens to improve research, or at least inform efforts to improve research through the sometime experimental evaluation of research itself.

Ioannidis et al. [32] categorised metaresearch into five major areas of interest: methods, reporting, reproducibility, evaluation and incentives (see Table 1), with this initial framework being used to facilitate action towards improving the transparency, reproducibility and efficiency of scientific research [33].

**Table 1:** The five major areas of metaresearch described by Ioannides et al. [32], with a non-exhaustive list of contemporary examples from the sport science or sports medicine literature

Types of metaresearch	Current examples in sport
Methods: ‘performing research’—study design, methods, statistics, research synthesis, collaboration, and ethics	Evaluations of flawed statistical methods in sport [34]; assessments on characteristics of study design such as experimental power [13–15]; handling of missing data [21] and questionable research practices in reporting of positive outcomes in sports medicine [18, 19]
Reporting: ‘communicating research’—reporting standards, study registration, disclosing conflicts of interest and information to patients, public and policymakers	Potential publication bias when reporting outcomes from experimental sport science research [20]; reporting and transparent research practices in sports medicine [24]; open science practices (inclusive of pre-registration, conflict of interest disclosure and funding information) in physical activity behaviour change intervention research [23] and authorship practices in sport science journals [35]
Reproducibility: ‘verifying research’—sharing data and methods, repeatability, replicability, reproducibility and self-correction	Reproducibility of methods (i.e., calculation of statistical power) [16]; proportion of articles within exemplar sport science journals that share data and/or code [36]; replication concerns relative to selected methodological issues in sport science [17]; frameworks for study selection for replication [37]; examples of study replication in sport science [38, 39]
Evaluation: ‘evaluating research’—pre-publication peer review, post-publication peer review, research funding criteria and other means of evaluating scientific quality	Post-publication evaluation of article retractions in sport science and sports medicine [40], investigation of option for open access selection prior to peer review in sport science literature [41]; development of guidelines for systematic reviewers in sport and exercise medicine for study evaluation after publication [42]
Incentives: ‘rewarding resear*c*h’—promotion criteria, rewards, and penalties in research evaluation for individuals, teams and institutions	Citations in Google Scholar profiles of sport science researchers [43]; incentives and consequences underpinning sport science research (including volume of publications and associated metrics) [44]; issues in the use of bibliometrics to supplement research evaluations (i.e., scholar promotions) in sport science [45]

Beyond these five areas, other frameworks have been developed to extend the application of metaresearch, such that individual metaresearch projects form part of a broader effort to continuously calibrate the scientific ecosystem toward higher standards of efficiency, credibility, and quality.

One example is the construction of a translational framework [46], designed to guide better metaresearch practice through principles centred on (a) identifying metaresearch problems, (b) investigating these problems, (c) developing solutions, and (d) evaluating solutions [46]. This framework aims to build an evaluative process around metaresearch, beginning with conceptual development and theoretical argumentation around identifying problems through to generating potential solutions. Potential solutions may involve developing new infrastructure, policy changes by key stakeholders such as universities, journals, or funders, and evaluating metaresearch-driven interventions on the research and scientific community.

Specific examples exist in other research fields for using metaresearch to develop and evaluate solutions. One well-known example relates to the idea of preregistration and registered reports. Pre-registration and registered reports aim to tackle the issue of questionable research practices (such as *p*-hacking and HARKing, see [47]), with these questionable practices being identified through metaresearch in the phases related to identifying and investigating problems. Initiatives such as preregistration and registered reports can assist with identifying selective reporting, by comparing the protocol and the report [48].

In the context of trying to improve openness and transparency in the peer-reviewed scientific literature, Nuijten et al. [49] noted a substantial increase in data availability from 8.6% to 87.4% of articles after a new policy at the journal *Judgement and Decision-Making* was implemented. This policy asked authors to publicly share data prior to article publication. To extend upon this, in the context of reporting guidelines, an early impact assessment of the Consolidated Standards of Reporting Trials (CONSORT) guidelines showed improvements in completeness and transparency of published reports [50] (e.g. categories such as “*unclear description of allocation concealment*” significantly decreased after a policy intervention).

It is important to acknowledge that metaresearch-informed interventions are not guaranteed to ensure research practice change; there are some examples where interventions designed to improve practice were shown to have poorer success. One example of this is from Nuijten et al. [49], who assessed data sharing in *Journal of Behavioural Decision Making* (different to the aforementioned example), with changes in data sharing remaining negligible across the same period (pre-open data policy, 0%; post-open data policy, 1.7%). Nevertheless, using metaresearch to inform and shape interventions is still necessary to ensure that any new initiative is constructed relative to evidence of a research practice problem.

With the growth of metaresearch broadly across scientific areas, some journals have created new sub-sections centred on metaresearch. This includes the integration of a Methodology and Research Practices section in the journal *Collabra: Psychology* [51] for psychological research, a new metaresearch section in *PLOS Biology* [52], a new translational meta-science collection in the journal *Clinical Science* [53] for clinical research, and the development of specific initiatives designed to reduce publication bias such as Registered Reports [54] in the journal *Cortex*. Additionally, new journals like *Meta-Psychology* have been established specifically for metaresearch in psychology [55].

This growth and interest in research aimed at improving scientific practice has also led to the development of specific centres and societies (see Table 2 for a non-exhaustive list of examples). These centres/societies focus on understanding, evaluating, and improving research practice, with some being both broader and research area ‘agnostic’ (e.g., Centre for Open Science [56]), others being specific to sports science and medicine (e.g., Society for Transparency, Openness, and Reproducibility in Kinesiology [57]) and smaller centres that exist within specific academic institutions [58]. Outcomes from metaresearch can also drive the generation of initiatives within these centres, which aim to improve the practice of conducting and producing research.

**Table 2:** A non-exhaustive list of examples of centres and societies that focus on improving scientific quality, often through metaresearch

Centre	Description
Centre for Open Science (COS) https://www.cos.io/	The Centre for Open Science (COS) is an international centre, consisting of scientific members across countries and fields of research. The COS is responsible for maintaining the Open Science Framework (OSF), which assists researchers in conducting rigorous and robust research, while also providing a platform to share work openly. The COS is also involved in conducting metaresearch to understand areas of inefficiency, and to evaluate interventions designed to improve research practice
Association for Interdisciplinary Metaresearch and Open Science (AIMOS) https://aimos.community/	The Association for Interdisciplinary Metaresearch and Open Science (AIMOS) is an Australian community which seeks to advance the interdisciplinary field of metaresearch by bringing together and supporting researchers conducting research in the field of metaresearch
Society for Transparency, Openness, and Reproducibility in Kinesiology (STORK) https://storkinesiology.org/	The Society for Transparency, Openness, and Replication in Kinesiology (STORK) provides a platform for kinesiologists, movement scientists, sport and exercise scientists, physical activity, and health scientists to collectively improve methods and practices or research and science within disciplines of kinesiology (i.e., sport science and medicine). Additionally, one of the 5 aims of the Society is to encourage metascience and critical evaluations of research practices within kinesiology
Sport Science Replication Centre https://ssreplicationcentre.com/	The Sports Science Replication Centre (based in Dublin, Ireland) has the intention to create a global, collaborative investigation into the replicability of research findings specific to the field of sports science using objective and transparent replication studies of current evidence
Society for the Improvement of Psychological Science (SIPS) https://improvingpsych.org/	The Society for the Improvement of Psychological Science (SIPS) is a psychology membership society founded to further promote improved methods and practices in psychological research. The Society aims to enhance the training of psychological researchers through promotion of research cultures that facilitate better quality research. SIPS also aims to quantify and empirically assess the impact of initiatives designed to improve research practice
Metaresearch Innovation Centre at Stanford (METRICS) https://metrics.stanford.edu/	The Metaresearch Innovation Centre at Stanford (METRICS) is a “*research-to-action*” centre, driven from within Stanford University, dedicated to finding ways to improve the validity and transparency of scientific research. METRICS draws on multi-disciplinary research collaborations in designing potential solutions that increase the effectiveness and value of research
Metaresearch Centre: Tilburg School of Social and Behavioural Sciences https://metaresearch.nl/	The Metaresearch Centre at Tilburg University is a university-driven research group at Tilburg University focused on metaresearch, specifically in psychology. Common areas of interest within the centre include statistical power and interpretation of statistical results, the use of questionable research practices, errors in publications, pre-registration, and replications
MetaMelb Research Initiative https://arts.unimelb.edu.au/school-of-historical-and-philosophical-studies/our-research/metamelb	The MetaMelb Research Initiative is an interdisciplinary research group, based at the University of Melbourne, focused on metaresearch and meta-science investigations
Centre for Journalology https://ohri.ca/journalology/	Journalology is the science of publication practices and the study of these activities. The Centre for Journalology is based in the Methods Centre of the Ottawa Hospital Research Institute (OHRI). The goal of the centre is to help enhance the reporting quality of research in order to increase the value of biomedical research

## The Importance of Performing Metaresearch, and Doing it Well

Some researchers may mistakenly believe that performing metaresearch is easy, as most data involved are already available in the form of research outputs, researcher profiles, citations metrics and other publicly available forms of meta-data. Additionally, there may also be perceptions that the act of conducting a replication could be viewed as dishonest or not even research, given that a researcher conducting a replication is not testing a novel hypothesis, or contributing meaningfully to science within their discipline. Despite this, metaresearch as a discipline is extremely difficult, given that it requires the careful critical lens often used in science being applied to science itself, leading to a risk that metaresearch can suffer similar problems to other fields of research and be an additional contribution to research waste if it is not performed well.

Research waste is evident in other data synthesising activities, such as meta-analyses and systematic reviews, which have grown in recent years. Despite the importance of meta-analyses and systematic reviews, many are low-quality or redundant, thus contributing to research waste [59–61]. It has been stated, and appears logical, that when done properly, evidence synthesis approaches such as meta-analyses are time-consuming and methodologically complex [62]. The data extraction and treatment processes also often require more attention to detail than clinical studies [62]. Evidence suggests that many meta-analyses are performed poorly, leading to problems, including within sports science. For example, a recent report showed that a substantial proportion (85%) of the 20 most highly cited meta-analysis studies in strength and conditioning had methodological errors [63]. Common errors included the calculation of effect sizes using standard error rather than standard deviation, and a failure to account for correlated observations through modelling approaches. None of these errors in the 20 meta-analyses, unfortunately, were detected during the peer-review process, possibly reflecting a generalised lack of knowledge of meta-analytic methods. Additionally, these types of errors have the potential to have large consequences, given the impact that they have on conclusions and any decisions associated with them. More recently, Borg et al. has also highlighted a tendency for misinterpretation of random effect meta-analyses in sport research, which could lead to potentially harmful treatments, or those lacking a sufficient evidence base, being used in practice [64].

More broadly in health and medical research, systematic evaluations of metaresearch studies have revealed issues in reporting information, with few metaresearch studies reporting enough information for verification or replication [65].

To prevent research waste, a high level of planning and consideration must be applied when undertaking metaresearch studies to ensure that outcomes align with the intended goals of performing metaresearch (e.g., policy changes). This would likely require undertaking metaresearch in teams that have individuals with the necessary skills for conducting metaresearch (i.e., input from team members with appropriate statistical or methodological training and expertise, team members with a strong understanding of theory and how it connects to application and practice, etc.). This also could extend to team members with computational skills sets, to enable metaresearch to be performed at scale, relying on automation rather than manually screening research items as a part of the data synthesising process. The potential of this approach has been highlighted in relation to the peer-review processes for journals, where different tools could be used to identify beneficial practices and common problems in preprints or submitted manuscripts, given the exploration of tools for checking statistical errors and other characteristics of experimental design (e.g., sample size estimation) by some publishers [66].

Additionally, Jager and Leek [67] have demonstrated the power of integrating text mining to facilitate metaresearch, through the use of a text extraction algorithm to estimate the rate of false discoveries in the medical literature, by using reported *p*-values as the data. One consideration for using automated methods of data extraction in metaresearch is the lack of common reporting for these types of studies. This is despite initiatives such as the Enhancing the QUAlity and Transparency Of health Research (EQUATOR) Network to assist researchers in improving the quality of scientific reporting [68]. Drawing on these guidelines is essential for ensuring that metaresearch does not contribute to research waste.

Another important consideration of metaresearch is that the tools and methods being applied have robust theoretical underpinnings, have been validated and that any limitations are acknowledged. As an example, when analysing *p*-values across research studies there can be problems when trying to accurately synthesise and infer meaning from these as a form of data, due to the difficulty in extracting required information from full-text articles using automated methods. Further to this, Mesquida et al. [69] also highlighted potential issues with automating the extraction of information like *p*-values from articles, with studies often testing vague and/or multiple hypotheses and the presence of multiple dependent variables leading to information being extracted which is not representative of the original experimental goals. This has led to a reliance on manually checking articles prior to extracting information.

The *p*-curve [70] has been used frequently in metaresearch and examines the distribution of significant *p*-values from a set of studies to determine whether the findings contain evidential value and thus increase our prior belief that a specific hypothesis is true and there is a non-zero effect. Examples of *p*-curves have been demonstrated across different scientific disciplines [71], including sport [69], with this serving as empirical evidence that *p*-hacking is potentially widespread in the scientific literature. Despite this, the *p*-curve has been shown to have problems. For example, even with minimal omitted-variable bias (e.g., unaccounted confounding), it is difficult to distinguish between *p*-curves based on true effects and *p*-curves based on null-effects with *p*-hacking [72]. Violations of randomisation in experimental studies can also result in situations where *p*-curves are unreliable [72]. Additionally, some criticisms of *p*-curves have been made, relating to the *p*-curve drawing on a model for the observed data identical to that of Hedges [73], yielding upwardly biased and highly inaccurate estimates of the population average effect size when effect sizes are heterogeneous [74]. This discussion around *p*-curves highlights that when using specific tools, metaresearch itself is not immune to criticism, and requires critique and careful administration of processes for better understanding of scientific behaviour. Like most areas of science, partnering with both domain-specific and methodological experts, and adopting a multi-faceted approach to exploring concepts such as bias (i.e., by using a range of tools [75], can maximise the value obtained from metaresearch studies.

Furthermore, the fact that a sort of ‘meta-metaresearch’ is already emerging [75] should not, in our opinion, be seen negatively but rather as a positive consequence of the research process which includes the scrutiny of peers.

## Getting Started with Metaresearch

### Beginning Early

It is important to ensure metaresearch is conducted early within fields of investigation. If large bodies of evidence are published across decades without scrutiny, it may become too difficult to recalibrate claims based on poor research. This can lead to literature populating potential ‘null fields’ across sport research where any effects are likely to be due primarily to bias, in a similar way to how this has been demonstrated in other fields (e.g., homeopathy [76]). In sport, different treatments and interventions can be implemented before reasonable supporting evidence exists, because of the ‘slow’ speed of research. The speed at which preliminary work is translated to practice in applied sport is often fast, as the outcomes of experiments can be implemented into practice quickly by athletes or teams seeking a competitive advantage [77]. This can be problematic as methods that lack a sufficient evidence base result in lost labour and resource waste, and lead to poorly supported research being implemented too quickly into practice. An example of this in sport is the rapid and widespread adoption of the acute-chronic workload ratio (ACWR) into professional practice, despite there being insufficient evidence supporting it, or even strong evidence refuting its efficacy [78–81].

There are other examples of research outcomes being rushed into practice in sport, including testosterone and its links with performance. The IAAF presented work by Bermon and Garnier [82] which measured the correlation between podium performances in athletes and levels of testosterone in athletics. The authors found a recruitment bias of women with disorder of sex development (DSD) in specific athletic events. A causal relationship between testosterone levels and athletic performance among women was then assumed, with this information influencing decision making and policy for athletes in these events. Follow-up work from Pielke et al. [83] pointed towards problematic issues in this research. When Pielke et al. asked for the data to reproduce the results, they were only granted 25% of the sample. Despite lacking the complete set of data, Pielke et al. concluded that Bermon and Garnier’s study was based on “significant flaws leading to unreliable results” and that “scientific integrity” was compromised. On 17 August 2021 (after the conclusion of the Tokyo Olympics), a correction by Bermon and Garnier [84] was published. In the correction, the original researchers admitted that the claims of a *causal* relationship between testosterone levels and athletic performance among female athletes in the restricted events was incorrect.

It is important to acknowledge that metaresearch itself is a reflexive process, and that investment in other activities designed to improve the quality of research outcomes is also important for the field to progress, for example, adopting ‘Big Team’ approaches [85] for conducting high quality generalisable research. Despite this, metaresearch can still be viewed as an important activity that assists in not just monitoring possible problems emerging in sport science and medicine, but by organising information in a way that makes it easier to connect with other fields (i.e., statistics, data science, epidemiology, philosophy, sociology, etc.) to ensure that not only are problems flagged early, but fit for purpose suggestions for how to change are also introduced early.

### Learning from Other Fields

There are also lessons that sport can learn from common errors and mistakes that have been made in other fields. Given the breadth of problems in sport at present, there are many examples that could warrant specific attention in sport. For example, Hoekstra et al. found that a non-statistically significant finding was misinterpreted as evidence of no effect in more than half of a sample of published articles in psychology, and in about 20% of the articles, a significant finding was considered absolute proof of the existence of an effect [86]. It is reasonable to assume that the same issues with *p*-value misinterpretation are common in sport science research. Similarly, in medicine, research exploring problems associated with common study designs has uncovered a broad range of potential issues, including the influence of commercial sponsorship on research design and outcomes, and poor research utility (e.g. having a real problem to fix, appropriate anchoring of the question within the context of prior evidence, etc.) and multiplicity issues (e.g. assessing the primary endpoint in more than one way) [87], many of which are highly likely to be present in sport research, but have been largely unexplored. Additionally, given the already highlighted issues with meta-analyses in sport [63], it is also important that the lack of validity and reproducibility of meta-analyses should be systematically explored.

There are common problems in health research that are also likely to be present in sport but which have been unexplored. For example, in 1659 randomised clinical trials, 62% of trials presented a high risk of bias and 30% were unclear in terms of the amount of bias, with over 220,000 participants being in poor quality trials [88]. Given the prevalence of bias in health trials and the similarities in approach to research between health and sport, this also presents as a problem worth investigating for sport. Other examples of areas that have been investigated in medicine but not sport include the extent of effect size exaggeration in randomised controlled trials due to small sample sizes combined with publication bias [89] (inclusive of reliability studies [90]) and the misappropriation of causal language in applied research [91], with these issues already being raised in sport medicine research [92].

Nefarious research practices have also been highlighted across research areas, with one study reporting the manipulation of data being common among scientists, 2% of scientists self-reporting having falsified research at least once, and 34% of scientists admitting to other questionable research practices [93]. At the publisher level, independent of research area, journal editors and scientists are often more interested in positive (significant) findings than negative findings, and in tests of new ideas rather than work verifying or refuting previous findings. This attitude inevitably leads to publication bias since trials with a statistically significant result are more likely to be published than those with a nonsignificant result [94]. All of these factors are clear within other research domains, or across all research fields, and present useful starting points for being able to understand the breadth of problems in sport science and sport medicine research.

## Broad Considerations for Using Metaresearch

### Interdisciplinary Expertise

Metaresearch should ideally be carried out by multi-disciplinary research teams with the range of skills required to address the problem or question being asked. Such partnerships could also lead to the development of new tools and software that help to further progress the development and quality of metaresearch (e.g., [66]). These interdisciplinary partnerships could also lead to novel development activities for graduate students and early career researchers in terms of ‘how’ to conduct metaresearch. Such training programs exist and have been piloted in areas outside of sport, such as in the biological sciences [95].

### Formalised Embedding Within Disciplines of Sport Science and Medicine

Metaresearch should be prioritised by journals, perhaps similarly to the way that other fields have created sub-sections or special issues in sport science and sports medicine. Additionally, rather than performing systematic reviews and meta-analyses across all research topics at the beginning of a graduate student journey within a thesis, critical evaluations of research could also be used to form the introduction, framing and basis of graduate student topics, inclusive of conducting replications. This would minimise research waste and provide robust information on the utility of information within a certain research area.

### Advocating for Better Infrastructure and Guidelines to Permit Better Metaresearch

Sport science and sports medicine should continue moving towards an improvement in reporting quality and completeness of information in research articles (i.e., open science practices, data availability, code sharing, reporting of methods, etc.), which in turn will help improve the types of metaresearch that are possible in sport science and sports medicine. Sport could also move towards adopting or adapting established guidelines for reporting of research within articles. As one example, the EQUATOR Network should be drawn upon as a standard for reporting practices in sport science research [68]. Similarly, the Checklist for statistical Assessment of Medical Papers (CHAMP) statement could be drawn on to improve reporting of methods and statistical practices [96]. Infrastructure changes in sport could also extend to the development of society- or domain-wide digital and web-based infrastructure for improving practice, including registries (similar to clinical registries) for consolidation of data and research information. This would allow for more well-designed accessible online systems, with design of meta-data for performing metaresearch. Additionally, national and international organisations, societies and funding bodies of sport research could provide avenues for metaresearch to be funded as a part of existing schemes.

### Ethics in Metaresearch

Different jurisdictions maintain unique regulatory frameworks for conducting metaresearch, including meta-analyses and systematic reviews. As secondary research, metaresearch typically relies on existing data, often not necessitating Human Research Ethics Committee review. However, different requirements exist concerning the use and access of previously collected data. While an ethics review may not be required, some institutions may issue a declaration ensuring that data usage and storage meet research expectations. This becomes particularly significant when metaresearch involves soliciting and collecting data from original data owners. Pre-registration of metaresearch studies could assist in providing transparency around methods to be used in metaresearch, allowing critique and review of the methods to be used prior to undertaking a metaresearch study. This would in turn lead to metaresearch that is conducted with integrity.

### Using Ethical Principles to Guide Metaresearch

Metaresearch aligns with a core principle in human research ethics review: to ensure the appropriate use of participant data. Metaresearch helps achieve this goal by evaluating the quality of research methods and findings, the interpretations drawn from them, and their subsequent influence on policy and practice. To guide metaresearch inquiries, an ethical framework can be beneficial. Adopting a perspective of questioning “what we might take for granted” [97] allows for a critical review of prevailing conclusions and practices, advancing scientific knowledge. As publication rates in sports science increase, dominant narratives may emerge without sufficient discourse. Metaresearch rooted in Foucauldian ethics can counteract this trend by evaluating prevailing narratives and questioning the quality and accuracy of what may become accepted in the literature [98].

### Metaresearch Can Complement Other Initiatives for Improving Science

Although metaresearch can provide an evidence base for the state of scientific quality and form the basis on which to frame interventions designed to improve science, alone it will not solve all problems associated with scientific quality. There are well established arguments for there being ‘theory crises’ in other fields such as management research [99] and psychology [100], with these relating specifically to experiments and research being built on the back of poorly framed theories (or no theory at all). Although the issue of poor theories underpinning research is something independent of many empirical metaresearch goals, some of the suggestions for how to improve theory building in research involve integration of metaresearch concepts, such as focusing on reproducibility [100], demonstrating that metaresearch may have a role in supporting some of these initiatives.

To build on issues related to theory, one reviewer commented that “meta-research efforts are not important if studies do not answer important, thoughtful questions (the ‘what’ and ‘why’ of research).” The investigation of important and meaningful questions is integral for conducting high quality research in sport, and the quality of research questions is also likely to be affected if the theory underlying such questions is considered to be poor. Although not central to the focus of the current paper, developing frameworks and principles for the tracking and evaluation of quality research questions would also work in conjunction with metaresearch for improving science more generally in sport.

There have also been suggestions that there is likely to be difficulty assessing the quality of research questions (and quality of research more broadly), but with an acceptance that this is still a difficult task to perform given that there are challenges making research quality a grounded theoretical concept [101]. Complexities surrounding the assessment of research quality can relate to the issue that research quality may require some level of consideration at the discipline level (i.e. particular research field or domain) [101], while similarly requiring a richer understanding of how to assess the quality of core components of research such as hypotheses [102]. As such, the assessment of research quality could definitely feature as a future part of metaresearch in sport but would require more thought in defining how quality should be assessed.

## Conclusion

Metaresearch is a needed sub-field within sport science and sports medicine, particularly given the evidence of poor research quality from work already conducted in sport. However, attempts to evaluate and correct practice in sport science and medicine through metaresearch should be undertaken with care and consideration, to ensure that metaresearch itself does not contribute to research waste. Specific consideration should be given to leveraging knowledge and expertise from other domains and disciplines, beginning metaresearch early to stop poor practice becoming normalised, and undertaking metaresearch ethically.

Finally, metaresearch is certainly not a solution to all problems in sport science and medicine research, and there are aspects still under debate that will need to be addressed in the future [103]. Furthermore, as one of the reviewers correctly pointed out, good methods do not make an irrelevant study relevant. Nevertheless, metaresearch can be a useful post-publication formal process contributing to identifying major problems in the field and, consequently, to finding solutions.

## Declarations

### Data availability

No data were used in this article.
